# Efficacy of Life Review Therapy Intervention on Life Satisfaction Among Older Adults Living in Taiwan Nursing Homes

**DOI:** 10.1093/geroni/igaa057.1230

**Published:** 2020-12-16

**Authors:** Ching-Teng Yao, Chia-Ju Lin

**Affiliations:** Kaohsiung Medical University, Kaohsiung, Taiwan (Republic of China)

## Abstract

Life review therapy, used as part of a comprehensive therapy plan for increasing the quality of life of the elderly, helps them to resolve their past conflicts, and accept their present conditions. This study tested the effectiveness of a structured life review therapy protocol on the life satisfaction of institutionalized older adults. A quasi-experimental design was adopted in this study. Fifty older adults aged 65 or above were recruited from nursing homes in southern Taiwan through convenience sampling. The participants in the intervention group carried out life review therapy for eight weeks in addition to their daily activities. The participants in the comparison group maintained their daily activities. Both groups were evaluated using a life-satisfaction scale including two aspects of life worries and situations in weeks 1 and 8. Data were collected at baseline (T1), immediately post-intervention (T2). Generalized estimating equations were used to examine the effect of the intervention on the outcomes. The overall life satisfaction increased significantly over time for the intervention group compared to the comparison group from week 1 to week 8. The life review therapy programs showed promising effects in improving the life satisfaction of older adults living in nursing homes.

